# Supplementary material for: Non-Invasive MRI of Blood–Cerebrospinal Fluid Barrier Function
Source: Nat Commun. 2020 Apr 29;11:2081. doi: 10.1038/s41467-020-16002-4 (PMC7190825; doi:10.1038/s41467-020-16002-4)
Supplement: Supplementary file 1 — Supplementary Information [file 41467_2020_16002_MOESM1_ESM.pdf]

Supplementary Figures for:

Non-Invasive MRI of Blood-Cerebrospinal Fluid Barrier Function

Evans *et al.,*

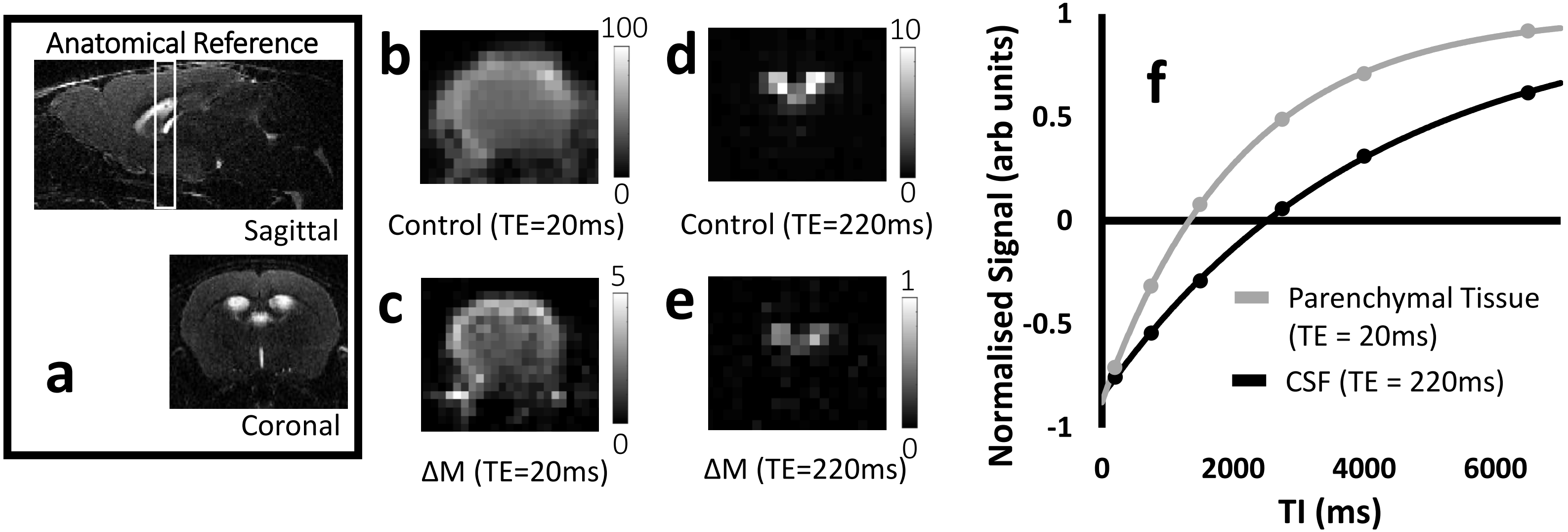

supplementary Figure 1

a. Anatomical reference images showing the location of the imaging slice at the caudal section of the lateral ventricles
b. Control image (standard ASL, TE = 20 ms)
c. Subtraction (ΔM) image (standard ASL, TE = 20 ms)
d. Control image (BCSFB-ASL, TE = 220 ms)
e. Subtraction (ΔM) image (BCSFB-ASL, TE = 220 ms)
f. Control signal as a function of TI with T1 inversion recovery fit (used to calculated tissue/CSF T1 and M0) for a representative mouse

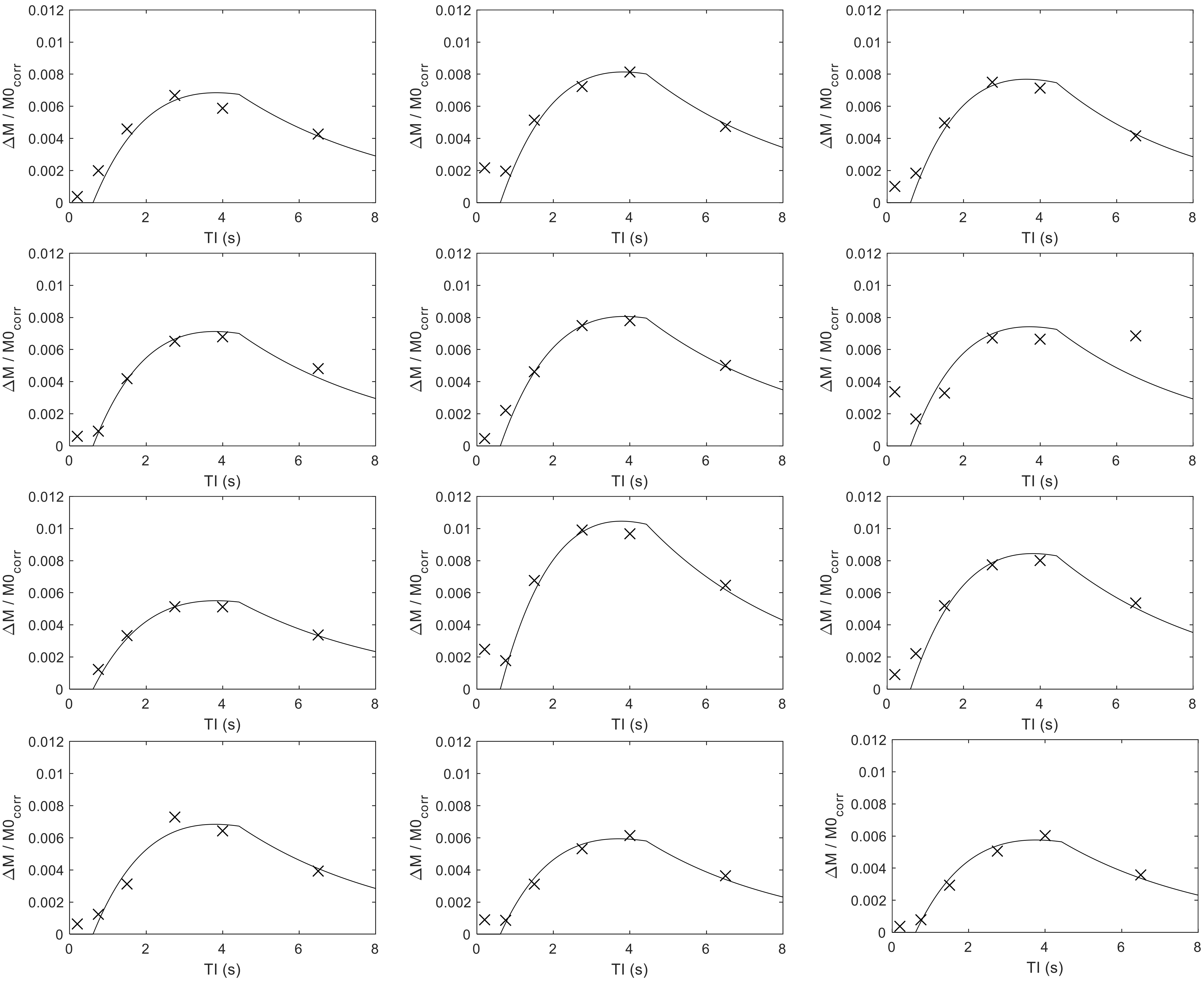

supplementary Figure 2

Normalised BCSFB-ASL signal as a function of TI (crosses) together with the adapted kinetic ASL model fit to the data (black line). Each plot shows the data from each of the individual 12 mice ('Multi-TI BCSFB-ASL in the Lateral Ventricles').

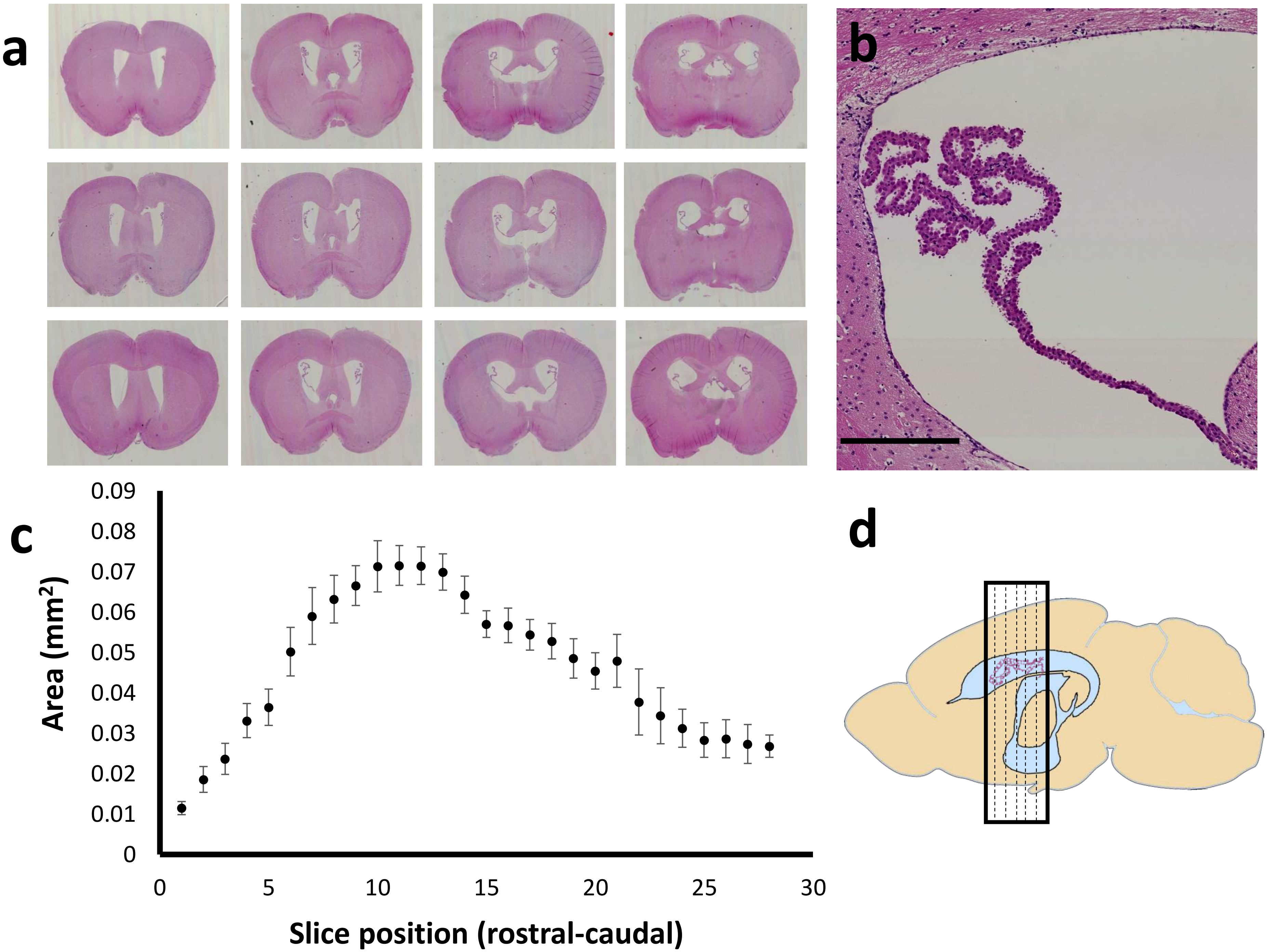

supplementary Figure 3

a) Example slices through the lateral ventricles from rostral to caudal (columns 1-3) for three example mice (rows 1-3). A total of n=11 biologically independent samples was examined with similar results.

b) Example 'zoomed in' image of the lateral ventricles, used to segment the CP tissue to calculate the area within each slice. A total of n=11 biologically independent samples was examined with similar results. Scale bar represents 200µm.

c) The estimated CP area across the lateral ventricles from the rostral to caudal slices (error bars represent the SEM, n=11 biological independent animals examined over 12 independent experiments for each group respectively)

d) Schematic illustrating the approximate location of the brain slices taken for which CP area was calculated.

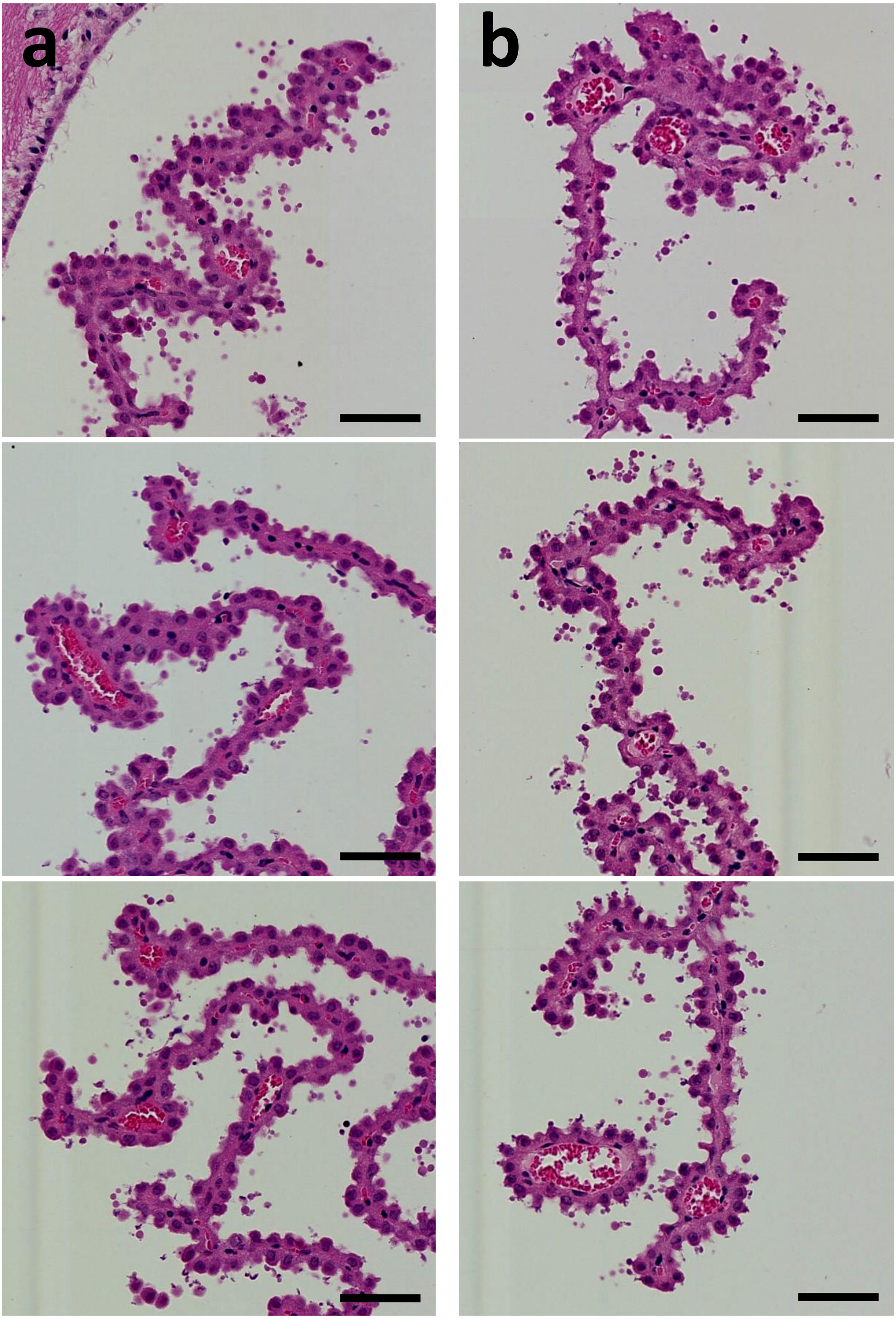

supplementary Figure 4

Example sections of choroid plexus tissue in the lateral ventricles in adult (column a) and aged mice (column b). Scale bar = 50µm. A total of n=11 and n=6 biologically independent samples was examined with similar results for the adult and aged mouse brain respectively.

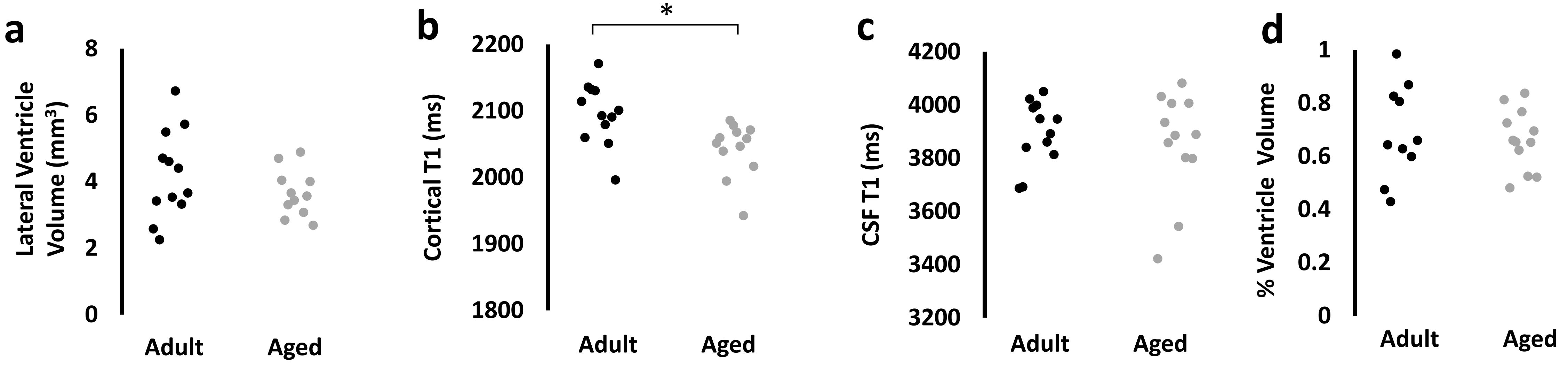

supplementary Figure 5

Estimates of the lateral ventricle volume (a), cortical T1 (b) , lateral ventricle CSF T1 (c) and ventricle volume as a % of whole brain volume (d) for the aged and adult cohort. Each dot represents an individual mouse.

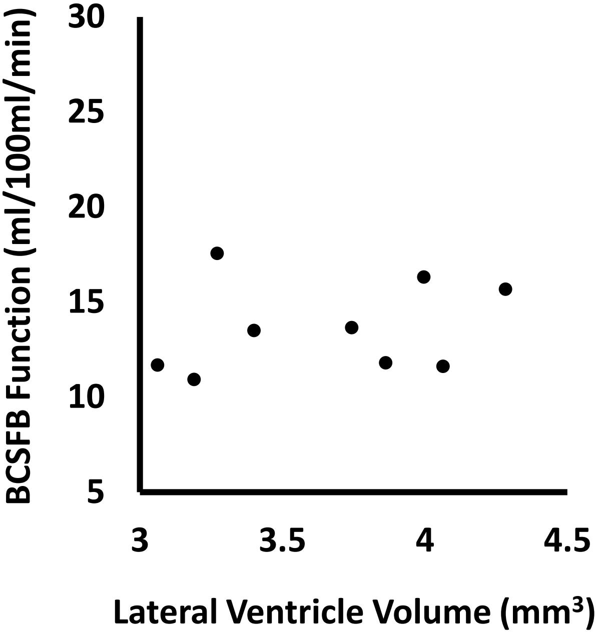

supplementary Figure 6

Rates of BCSFB-mediated blood water delivery to the ventricular CSF against the volume of the lateral ventricles for the separate cohort of aged mice (C57BL/6j, male, 24-25 months of age). Each dot represents each individual mouse (n=10).

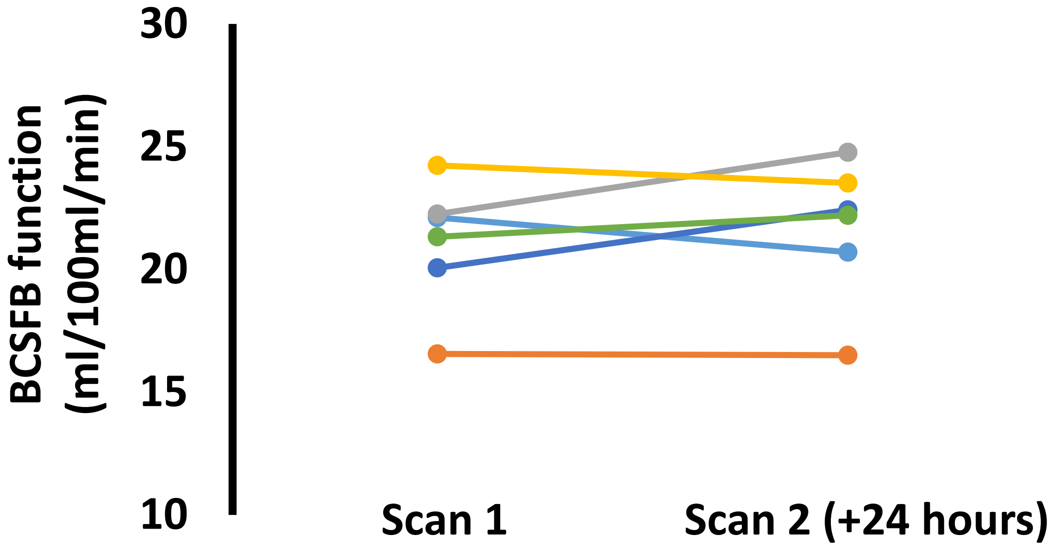

supplementary Figure 7

Estimate of BCSFB-mediated water delivery rates to the lateral ventricles at baseline (scan 1) and then 24 hours later following recovery. Each line represents an individual mouse.
