# Supplementary material for: Non-Invasive MRI of Blood–Cerebrospinal Fluid Barrier Function
Source: Nat Commun. 2020 Apr 29;11:2081. doi: 10.1038/s41467-020-16002-4 (PMC7190825; doi:10.1038/s41467-020-16002-4)
Supplement: Supplementary file 3 — Reporting summary [file 41467_2020_16002_MOESM3_ESM.pdf]

nature research

Corresponding author(s): Jack Wells

Last updated by author(s): 2020-17-03

Reporting Summary

Nature Research wishes to improve the reproducibility of the work that we publish. This form provides structure for consistency and transparency in reporting. For further information on Nature Research policies, see Authors & Referees and the Editorial Policy Checklist .

Statistics

For all statistical analyses, confirm that the following items are present in the figure legend, table legend, main text, or Methods section.

n/a	Confirmed	
☐	☒	The exact sample size (*n*) for each experimental group/condition, given as a discrete number and unit of measurement
☐	☒	A statement on whether measurements were taken from distinct samples or whether the same sample was measured repeatedly
☐	☒	The statistical test(s) used AND whether they are one- or two-sided<br>*Only common tests should be described solely by name; describe more complex techniques in the Methods section.*
☒	☐	A description of all covariates tested
☐	☒	A description of any assumptions or corrections, such as tests of normality and adjustment for multiple comparisons
☒	☐	A full description of the statistical parameters including central tendency (e.g. means) or other basic estimates (e.g. regression coefficient) AND variation (e.g. standard deviation) or associated estimates of uncertainty (e.g. confidence intervals)
☐	☒	For null hypothesis testing, the test statistic (e.g. *F*, *t*, *r*) with confidence intervals, effect sizes, degrees of freedom and *P* value noted<br>*Give P values as exact values whenever suitable.*
☒	☐	For Bayesian analysis, information on the choice of priors and Markov chain Monte Carlo settings
☒	☐	For hierarchical and complex designs, identification of the appropriate level for tests and full reporting of outcomes
☒	☐	Estimates of effect sizes (e.g. Cohen's *d*, Pearson's *r*), indicating how they were calculated

Our web collection on statistics for biologists contains articles on many of the points above.

Software and code

Policy information about availability of computer code

Data collection	The MRI data was collected using a 9.4T Agilent and a 3T Philips MRI scanner respectively. The histology data was collected on a Nanozomer microscope.
Data analysis	All the MRI data was analysed in Matlab 2018a. The histology data was analyzed using NPD view (Hamamatsu).

For manuscripts utilizing custom algorithms or software that are central to the research but not yet described in published literature, software must be made available to editors/reviewers. We strongly encourage code deposition in a community repository (e.g. GitHub). See the Nature Research guidelines for submitting code & software for further information.

Data

Policy information about availability of data

All manuscripts must include a data availability statement. This statement should provide the following information, where applicable:

- Accession codes, unique identifiers, or web links for publicly available datasets
- A list of figures that have associated raw data
- A description of any restrictions on data availability

The raw imaging data will be deposited on the UCL data repository:

https://www.ucl.ac.uk/library/research-support/research-data-management/ucl-research-data-repository

https://doi.org/10.5522/04/12037521.v1

Field-specific reporting

Please select the one below that is the best fit for your research. If you are not sure, read the appropriate sections before making your selection.

- [x] Life sciences
- [ ] Behavioural & social sciences
- [ ] Ecological, evolutionary & environmental sciences

For a reference copy of the document with all sections, see nature.com/documents/nr-reporting-summary-flat.pdf

Life sciences study design

All studies must disclose on these points even when the disclosure is negative.

Sample size	No sample size calculation was performed
Data exclusions	Of the 80 mice that were imaged in this study. Data from two were excluded, justification for which is described in the manuscript (methods).
Replication	Following the reviewers comments, we replicated the observed down-regulation in BCSFB function in the aged mouse brain in a separate cohort of aged mice. The new data is shown in supplementary Figure 6. We have since replicated the effect of vasopressin to down-regulate BCSFB function in several separate cohorts of mice (data not shown).
Randomization	The aged and adult mice shown in Figure 2 were imaged in an interleaved manner within ~ 3 days of imaging.
Blinding	The estimation of lateral ventricle volume was performed by an operator without knowledge of the corresponding functional data.

Reporting for specific materials, systems and methods

We require information from authors about some types of materials, experimental systems and methods used in many studies. Here, indicate whether each material, system or method listed is relevant to your study. If you are not sure if a list item applies to your research, read the appropriate section before selecting a response.

Materials & experimental systems

n/a	Involved in the study	
x		Antibodies
x		Eukaryotic cell lines
x		Palaeontology
	x	Animals and other organisms
	x	Human research participants
x		Clinical data

Methods

n/a	Involved in the study	
x		ChIP-seq
x		Flow cytometry
	x	MRI-based neuroimaging

Animals and other organisms

Policy information about studies involving animals; ARRIVE guidelines recommended for reporting animal research

Laboratory animals	Mice: C57BL/6 3 months female, C57BL/6j 6 months male, C57BL/6j 23 months male,C57BL/6j 24-25 months male.
Wild animals	na
Field-collected samples	*For laboratory work with field-collected samples, describe all relevant parameters such as housing, maintenance, temperature, photoperiod and end-of-experiment protocol OR state that the study did not involve samples collected from the field.*
Ethics oversight	All experiments were performed in accordance with the UK Home Office Animals (Scientific Procedures) Act

Note that full information on the approval of the study protocol must also be provided in the manuscript.

Human research participants

Policy information about studies involving human research participants

Population characteristics	3 participants. Male aged 26-35.
Recruitment	Recruited by asking my work colleagues. Possible recruitment bias not relevant to experiment hypothosis/aim.
Ethics oversight	UCL Hospital

Note that full information on the approval of the study protocol must also be provided in the manuscript.

Magnetic resonance imaging

Experimental design

Design type	NA - novel MRI method
Design specifications	NA - novel MRI method
Behavioral performance measures	NA

Acquisition

Imaging type(s)	NA - novel MRI method
Field strength	9.4T and 3T
Sequence & imaging parameters	NA - novel MRI method
Area of acquisition	NA - novel MRI method

Diffusion MRI ☐ Used ☒ Not used

Preprocessing

Preprocessing software	NA - novel MRI method
Normalization	NA - novel MRI method
Normalization template	NA - novel MRI method
Noise and artifact removal	none
Volume censoring	NA - novel MRI method

Statistical modeling & inference

Model type and settings	NA
Effect(s) tested	NA

Specify type of analysis: ☐ Whole brain ☒ ROI-based ☐ Both

Anatomical location(s) NA - novel MRI method

Statistic type for inference (See Eklund et al. 2016)	NA - novel MRI method
Correction	NA - novel MRI method

Models & analysis

n/a	Involved in the study
☒	☐ Functional and/or effective connectivity
☒	☐ Graph analysis
☒	☐ Multivariate modeling or predictive analysis
